# Difference in the Sexual and Reproductive Health of Only-Child Students and Students With Siblings, According to Sex and Region: Findings From the National College Student Survey

**DOI:** 10.3389/fpubh.2022.925626

**Published:** 2022-07-08

**Authors:** Shuangyu Zhao, Yun Liang, Jia Yi Hee, Xinran Qi, Kun Tang

**Affiliations:** ^1^Vanke School of Public Health, Tsinghua University, Beijing, China; ^2^School of Pharmaceutical Science, Tsinghua University, Beijing, China; ^3^Department of Epidemiology, Johns Hopkins Bloomberg School of Public Health, Baltimore, MD, United States

**Keywords:** only-child students, students with siblings, sexual and reproductive health, sexual attitudes, sexually transmitted infections (STI)

## Abstract

**Objective:**

The differences in sexual knowledge, attitudes, behaviors, seeking behaviors for sex-related knowledge, and sexual and reproductive health (SRH) outcomes among only-child students and students with siblings in China, was examined for sex- and region- specific effects.

**Research Design and Methods:**

Data on 49,569 students from the 2019 National College Student Survey on Sexual and Reproductive Health, conducted across 31 provinces in mainland China was utilized. Multivariable regression and stratified analyses were employed to analyze the differences in sexual and reproductive health between only-child students and students with siblings.

**Results:**

Only-child students reported higher sexual knowledge, more liberal sexual attitudes, and fewer adverse SRH outcomes compared to those with siblings. Results were found to be influenced by sex and hometown region after controlling for socio-economic factors, parent-child relationship, and sexuality education.

**Conclusions:**

Female students with siblings who resided in rural regions were more likely to have poorer SRH compared to male only-child students who resided in urban regions. Comprehensive sexual education for students should aim to better include females and students from rural areas both offline and online, and public healthcare should offer subsidized consultations and contraceptives.

## Introduction

The rapid development of China over the past 40 years resulted in economic growth and increased globalization (1). Advancements in areas such as technology and communication provided an abundance of resources for students to mature more rapidly physically and psychosocially, resulting in earlier sexual maturity (2, 3). Furthermore, globalization increased the influence of Western populations in China, where sexual experiences in unmarried students are high (4), creating more liberal attitudes toward sex in Chinese students (5). Vuong and Napier (6) proposes that this is due to the “mindsponge” mechanism, where the minds of individuals may be reshaped after exposure to different cultures, leading to substantial cultural changes nationally (6). Sexual and reproductive health (SRH) issues are therefore further exacerbated (7), resulting in higher rates of unplanned pregnancies, abortions, and sexually transmitted infections (8). However, given the conservative nature of the Chinese population, SRH education remains deficient and although modest efforts have been made, a large majority of students in China particularly in rural regions, have not had formal SRH education or access to SRH services.

Sexual attitudes and behaviors in the Chinese population are also largely affected by the one-child policy, the rural-urban divide, and traditional beliefs. During the one-child policy implemented between 1979 to 2015 in China, most Chinese families were limited to one child each to reduce China's population growth that was straining available resources. To promote the policy, only-child families were granted concessions such as easier school enrollment, better employment opportunities, and better healthcare benefits (9). However, as a result of the policy and China's patrilineal family systems, son preference governed much of reproductive behavior in China, resulting in a male-biased sex ratio (10). A significant change, called the 1.5-child policy, was later made in 1984 to the one-child policy; under the 1.5-child policy, couples residing in rural regions were allowed to have a second child if their first was a female (11). By the end of 2015, the year that the one-child policy was abolished, there was an estimated 225 million only-child children in China (12). Studies have shown that these children tend to be given more attention and care from parents (13), have better educational opportunities and higher school achievements than children with siblings (14), suggesting healthier development.

In addition, despite the modernization of China, China continues to utilize a dual economy, resulting in significant gaps in income, infrastructure, and government services between urban and rural areas (15). Due to the economic disparity between rural and urban areas, more than 168 million rural residents have migrated internally to cities, resulting in more than one-third of all rural children having had been left behind by either or both parents (16). Left behind children reportedly have more health deficits and are at greater risk of unhealthy development (16) and as such, may have poorer SRH. Furthermore, traditional beliefs such as Confucianism, ingrained into the Chinese culture, instills the concept of male superiority over females. As such, sexual attitudes and behaviors such as pre-marital sexual activities are more tolerated in males than in females (17). The physical differences between males and females also influence sexual attitudes and behaviors (18).

Whilst Yan et al. (19) and Li et al. (20) have investigated attitudes toward premarital sex and sexual knowledge, attitudes, and practices among only-child students and students with siblings, these studies were not conducted nationally. Given that residence in rural or urban regions and sex of the child may affect only-child status as well as sexual knowledge, attitudes, behaviors, and SRH outcomes, this study aims to investigate the differences in sexual knowledge, attitudes, behaviors, and SRH outcomes between only-child students and students with siblings in China stratified by region and sex. As students are more likely to seek information online, our findings may provide policy makers with evidence-based findings to guide the design of internet-based learning materials, particularly for female students and students from rural areas.

## Materials and Methods

### Study Design and Participants

Data from the 2019 National College Student Survey on Sexual and Reproductive Health conducted by the China Family Planning Association (CFPA) and collected by the China Youth Network (CYN) was utilized for this study. The CYN is the largest youth volunteer organization in China that provides SRH education. A total of 241 institutions of higher education including key universities, ordinary universities, and colleges were selected from all 31 provincial-level administrative regions in China, after balancing for the type of university and vocational college. Although there are more recent survey data, the 2019 survey data was utilized as it was the most recent survey round that contains measures on only-child students. The methods of survey instrument development and study design were previously described in detail elsewhere (21).

Utilizing convenience sampling, the internet-based self-administered questionnaire was distributed to students through contact points from selected universities and vocational colleges. A total of 55,757 responses from 1,764 universities and vocational colleges were collected. Data collected included sociodemographic, attitudes toward sexual behaviors, knowledge of SRH, sexual history, and SRH outcomes. Respondents were included in final analyses if they (1) provided informed consent, (2) answered all questions and passed the consistency checks and logic verification, and (3) were aged between 17 and 24. A total of 49,569 participants were included in our final analysis. This study was reviewed and approved by the Institutional Review Board of Tsinghua University (IRB No. 20190083).

### Exposure

The main exposure of interest was whether the participant was an only-child or if they had siblings. The question “How many children are there in your home (including yourself)?” was used. Participants who answered “one” were considered to be an only-child and were coded as “1,” while those who reported more than one child in family were considered to have siblings and were coded as “0.”

### Outcomes

The outcomes of interest are sex-related knowledge, attitudes, behaviors, and SRH outcomes. Sex-related knowledge was measured using 9 questions on contraceptive use, acquired immune deficiency syndrome (AIDS), abortion/pregnancy, and sexually transmitted diseases (STI). Response options were “yes,” “no,” and “do not know.” Each positive answer was coded as “1,” while the negative answer or “do not know” were coded as “0.” Sex-related knowledge scores ranged from 0 to 9, with increasing scores indicating higher sex-related knowledge.

Attitudes toward sexual behaviors were measured using 5 questions on premarital sexual intercourse, having had sexual intercourse during university, one-night stand, and multiple sexual partners, and guilt of being sexually active. Response options were “strongly disagree,” “disagree,” “unsure,” “agree,” or “strongly agree.” Responses “agree” and “strongly agree” were classified as positive attitudes, coded as “1,” while “unsure,” “disagree,” and “strongly disagree” were classified as negative attitudes, coded as “0.”

Sexual seeking behaviors were measured using 3 questions: “Have you ever read erotic magazines or books?”, “Have you ever searched for pornographic information online?”, and “Have you ever searched for sexual knowledge online?”. Response options were “yes,” coded as “1” or “no,” coded as “0.” Sexual seeking behaviors scores ranged from 0 to 3, with increasing scores indicating higher sexual seeking behaviors.

Sexual behaviors, including risky sexual behaviors, were measured using 5 questions: “Have you ever practiced masturbation?”, “Have you ever had sexual intercourse (anal or vaginal sex)?”, “Have you ever had casual sexual intercourse (including hook-up experience, one-night stand, and prostitution)?”, “Have you ever had multiple sexual partners at the same time?” and “Did you use any contraceptives during your first sexual intercourse?”. Response options were “yes,” coded as “1” or “no,” coded as “0.” The frequency of contraception use during sexual intercourse was also measured. Response options were “never,” “seldom,” “almost half of the times,” “mostly,” and “each time.” Responses were re-categorized into use of condoms in most sexual intercourse (“almost half of the times,” “mostly,” and “each time”) or no use of condoms in most sexual intercourse (“seldom” and “never”).

Adverse sex-related outcomes were measured using 3 questions on unintended pregnancy, induced abortion, and a STI diagnosis. Response options were “never,” “ever, in the past one year” and “ever, more than one year ago.” Response “never” was classified as no and coded as “0,” while the other options were classified as “yes” and coded as “1.” Adverse sex-related outcomes scores ranged from 0 to 3, with increasing scores indicating higher adverse sex-related outcomes.

### Other Covariates

Data on age, sex, ethnicity, hometown region, school type, average monthly expenditure, sexuality education at school, self-rated parent-child relationship, parents' highest educational qualifications, and parent-child discussion relevant to sexual behaviors/contraception, and tobacco and alcohol use were also collected. These factors have been demonstrated by published literature to have impact on SRH outcomes or to contribute to the difference between the only-child students and students with siblings (22–24).

Age and self-rated parent-child relationship were analyzed as continuous variables; self-rated parent-child relationship scores ranged from 0 to 10 with higher scores indicating better relationships. The remaining variables were coded as categorical variables. Ethnicity was categorized into Han and ethnic minorities. Hometown region, defined as the place of residence before attending university or vocational college, was categorized into “urban” and “rural.” The average monthly expenditure was categorized into 0-999 RMB, 1,000-1,999 RMB and ≥2,000 RMB. School type was categorized into “college” or “university.” Highest parental educational attainment was categorized into “primary and below,” “middle school,” “high school,” and “college and above.” Sexuality education at school, parent-child discussion relevant to sexual behaviors/contraception, and tobacco and alcohol consumption were categorized into “ever” and “never.”

### Statistical Analysis

Continuous variables were described as mean ± standard deviation (SD), and categorical variables were described as proportion and percentage. Continuous variables were compared using independent sample *t*-test and categorical variables were compared using the chi-square test. Linear regression was utilized to examine the association between only-child status with sex-related knowledge. Logistic regression was utilized to examine the association between only-child status with sexual attitudes, sexual behaviors, and adverse SRH outcomes, stratified by sex and hometown region. The models were adjusted for age, sex, ethnicity, school type, average monthly expenditure, self-rated parent-child relationship, sexuality education at school, parents' highest educational qualifications, and tobacco and alcohol use. All statistical analyses were conducted using STATA/SE version 15.1 (College Station, Texas 77845, USA).

## Results

### Participants' Characteristics

The characteristics of only-child students and students with siblings are presented in Table 1. Only-child students were less likely to be females (58.98 vs. 69.71%), of ethnic minority (6.86 vs. 10.93%), residing in rural areas (8.33 vs. 27.94%), have an average monthly expenditure between 0 to 999 yuan (7.52 vs. 17.60%), have ever received sexuality education at school (55.29 vs. 57.72%), and to report better self-rated father-child relationship (7.12 vs. 7.34). However, only-child students were more likely to be attending university (79.46 vs. 64.27%), have parents with educational qualifications college and above (47.81 vs. 14.18%), have had parent-child discussions relevant to sexual behaviors and contraception (37.12 vs. 24.49% and 33.31 vs. 18.85%, respectively), have smoked (15.28 vs. 12.32%) and have consumed alcohol (46.39 vs. 34.5%).

**Table 1:** Socio-demographic characteristics of only-child students and students with siblings.

	**Total (*N* = 49,569)**	**Students with siblings (*n* = 34,017)**	**Only-child students (*n* = 15,552)**	***p*-value**
	***n* (%)**	***n* (%)**	***n* (%)**
**Age (years), Mean** **±SD**	19.79 ± 1.34	19.72 ± 1.29	19.95 ± 1.41	<0.0001
**Sex**				<0.0001
Male	16,684 (33.66)	10,305 (30.29)	6,379 (41.02)	
Female	32,885 (66.34)	23,712 (69.71)	9,173 (58.98)	
**Ethnicity**				<0.0001
Han	44,785 (90.35)	30,300 (89.07)	14,485 (93.14)	
Others	4,784 (9.65)	3,717 (10.93)	1,067 (6.86)	
**Hometown region**				<0.0001
Rural	10,801 (21.79)	9,506 (27.94)	1,295 (8.33)	
Urban/suburban	38,768 (78.21)	24,511 (72.06)	14,257 (91.67)	
**School type**				<0.0001
College	15,350 (30.97)	12,155 (35.73)	3,195 (20.54)	
University	34,219 (69.03)	21,862 (64.27)	12,357 (79.46)	
**Average monthly expenditure (RMB)**				<0.0001
0–999	7,158 (14.44)	5,988 (17.60)	1,170 (7.52)	
1,000–1,999	28,743 (57.99)	21,135 (62.13)	7,608 (48.92)	
≥2,000	13,668 (27.57)	6,894 (20.27)	6,774 (43.56)	
**Ever received sexuality education at school**	28,333 (56.96)	19,634 (57.72)	8,599 (55.29)	<0.0001
**Self-rated parent-child relationship (0**–**10), Mean** **±SD**			
Fathers	7.27 ± 2.33	7.34 ± 2.30	7.12 ± 2.41	<0.0001
Mothers	7.88 ± 2.03	7.87 ± 2.04	7.90 ± 2.01	0.21
**Parental highest educational attainments**				<0.0001
Primary school and below	6.033 (12.17)	5,421 (15.94)	612 (3.94)	
Middle school	18,235 (36.79)	15,182 (44.63)	3,053 (19.63)	
High school	13,043 (26.31)	8,592 (25.26)	4,451 (28.62)	
College and above	12,258 (24.73)	4,822 (14.18)	7,436 (47.81)	
**Parent-child discussion relevant to sexual behaviors**				<0.0001
Never	35,465 (71.55)	25,686 (75.51)	9,779 (62.88)	
Ever	14,104 (28.45)	8,331 (24.49)	5,773 (37.12)	
**Parent-child discussion relevant to contraception**				<0.0001
Never	37,976 (76.61)	27,604 (81.15)	10,372 (66.69)	
Ever	11,593 (23.39)	6,413 (18.85)	5,180 (33.31)	
**Tobacco consumption**				<0.0001
Ever	6,568 (13.25)	4,191 (12.32)	2,377 (15.28)	
Never	43,001(86.75)	19,826 (87.68)	13,175 (84.72)	
**Alcohol consumption**				<0.0001
Ever	18,979 (38.29)	11,765 (34.59)	7,214 (46.39)	
Never	30,590 (61.71)	22,252 (65.41)	8,338 (53.61)	

### Sex-Related Knowledge, Attitudes, Behaviors and SRH Outcomes Among Only-Child Students and Students With Siblings

Sex-related knowledge, attitudes, and behaviors of only-child students and students with siblings are presented in Table 2. Only-child students had on average, higher sex-related knowledge scores (4.55 ± 2.27 vs. 3.62 ± 2.27), were more agreeable to premarital sexual intercourse (40.26 vs. 22.70%), sexual intercourse during university (57.29 vs. 35.51%), one-night stands (11.34 vs. 7.30%), having multiple sexual partners (14.44 vs. 5.61%), and were less likely to feel guilty about being sexually active (13.13 vs. 15.94%).

**Table 2:** Sex-related knowledge, attitudes behaviors and SRH outcomes between only-child students and students with siblings.

	**Total, *n* (%)**	**Students with siblings, *n* (%)**	**Only-child students, *n* (%)**	**χ2**	***p*-value**
**Sex-related knowledge, mean (SD)**	3.91 (2.31)	3.62 (2.27)	4.55 (2.27)	-	<0.0001
**Sexual attitude**					
Agree with premarital sexual intercourse	13,985 (28.21)	7,723 (22.70)	6,262 (40.26)	1,625.20	<0.0001
Agree with sexual intercourse during university	20,989 (42.34)	12,080 (35.51)	8,909 (57.29)	2,072.50	<0.0001
Agree with one night stands	4,246 (8.57)	2,483 (7.30)	1,763 (11.34)	222.07	<0.0001
Agree with multiple sexual partners	4,152 (8.38)	1,907 (5.61)	2,245 (14.44)	1,084.10	<0.0001
Felt guilty of being sexually active	7,466 (15.06)	5,424 (15.94)	2,042 (13.13)	66.10	<0.0001
**Seeking behaviors for sex-related information**					
Ever read erotic magazines or books	16,969 (34.23)	10,563 (31.05)	6,406 (41.19)	487.29	<0.0001
Ever searched for pornographic information online	27,194 (54.86)	16,166 (47.52)	11,028 (70.91)	2,357.30	<0.0001
Ever searched for sexual knowledge online	29,524 (59.56)	18,151 (53.36)	11,373 (73.13)	1,731.90	<0.0001
**Sexual behaviors**					
Practiced masturbation	26,267 (52.99)	15,548 (45.71)	10,719 (68.92)	2,309.40	<0.0001
Ever had sexual intercourse (anal or vaginal)	10,606 (21.40)	6,075 (17.86)	4,531 (29.13)	806.83	<0.0001
Ever had casual sexual intercourse^a^	1,771 (16.70)	881 (14.50)	1,771 (16.70)	49.30	<0.0001
No condom use during first sexual intercourse^a^	1,639 (15.45)	1,064 (17.51)	575 (12.69)	46.23	<0.0001
No condom use during most sexual intercourse^a^	1,525 (14.38)	1,004 (16.53)	521 (11.50)	53.30	<0.0001
Have had multiple sexual partners^a^	4,466 (42.11)	2,497 (41.10)	969 (43.46)	5.90	0.015
**Adverse reproductive health outcomes**					
Ever had unintended pregnancy^a^	419 (3.95)	284 (4.67)	135 (2.98)	19.66	<0.0001
Ever had induced abortion^a^	378 (3.56)	252 (4.15)	126 (2.78)	14.12	<0.0001
Ever been diagnosed sexual transmitted infections	524 (1.06)	341 (1.00)	183 (1.18)	3.10	0.08

a *Excludes students who have never had sexual intercourse (included =10,606). The details are showed in Supplementary Table S1*.

Furthermore, only-child students reported higher sexual seeking behaviors such as reading erotic magazines or books (41.19 vs. 31.05%), and searching for pornographic information and sexual knowledge online (70.91 vs. 47.52% and 73.13 vs. 53.36% respectively). Only-child students also reported higher sexual behaviors, such as masturbation (68.92 vs. 45.71%), sexual intercourse (anal or vaginal sex) (29.13 vs. 17.86%), casual sexual intercourse (16.70 vs. 14.50%), and having multiple sexual partners (43.46 vs. 41.10%). However, only-child students reported lower risky sexual behaviors such as no condom use during first sexual intercourse (12.69 vs. 17.51%) and no condom use in most sexual intercourse (11.50 vs. 16.53%). As for adverse reproductive health outcomes, only-child students were significantly less likely to have had unintended pregnancies (2.98 vs. 4.67%) and abortions (2.78 vs. 4.15%).

### Associations Between Only-Child Students With Sex-Related Knowledge, Attitudes, Behaviors and SRH Outcomes, Stratified by Hometown Region and Sex

The associations between only-child status with sex-related knowledge, attitude, and behaviors, stratified by hometown region and sex, are presented in Table 3. Only-child students have on average, higher sex-related knowledge scores regardless of their hometown region (rural: beta 0.14, 95% CI 0.01–0.27; urban: beta 0.37, 95% CI 0.32–0.42) or sex (males: beta 0.24, 95% CI 0.17–0.31; females: beta 0.43, 95% CI 0.37–0.48), compared to students with siblings.

**Table 3:** Associations between only-child status with sex-related knowledge, attitudes, behaviors and SRH outcomes stratified by region and sex^†^.

	**Hometown region** ^**a**^		**Sex** ^**b**^	
**Variables**	**Rural**	**Urban/Suburban**	**Male**	**Female**
**Sex-related knowledge** ^**d**^	0.14 (0.01–0.27)*	0.37 (0.32–0.42)***	0.24 (0.17–0.31)***	0.43 (0.37–0.48)***
**Sexual attitudes**				
Agree with premarital sexual intercourse	1.15 (1.00–1.34)	1.34 (1.27–1.41)***	1.14 (1.06–1.22)**	1.47 (1.38–1.57)***
Agree with sexual intercourse during university	1.09 (0.95–1.27)	1.39 (1.32–1.46)***	1.17 (1.09–1.26)***	1.50 (1.40–1.58)***
Agree with one night stand	1.19 (0.97–1.47)	1.17 (1.08–1.27)***	1.03 (0.94–1.15)	1.35 (1.22–1.50)***
Agree with multiple sexual partners	1.13 (0.87–1.46)	1.51 (1.40–1.64)***	1.24 (1.11–1.38)***	1.69 (1.53–1.86)***
Felt guilty of being sexually active	0.93 (0.80–1.09)	0.85 (0.80–0.91)***	0.90 (0.82–0.99)*	0.81 (0.74–0.88)***
**Seeking behaviors for sex-related information**		
Ever read erotic magazines or books	1.01 (0.88–1.15)	1.16 (1.10–1.21)***	0.97 (0.91–1.04)	1.27 (1.20–1.35)***
Ever searched for pornographic information online	1.20 (1.04–1.37)*	1.55 (1.47–1.64)***	1.19 (1.10–1.29)***	1.70 (1.61–1.81)***
Ever searched for sexual knowledge online	1.06 (0.94–1.21)	1.47 (1.40–1.55)***	1.13 (1.05–1.22)**	1.63 (1.53–1.73)***
**Risky sexual behaviors**			
Ever had casual sexual intercourse^c^	1.69 (1.12–2.56)**	1.05 (0.93–1.19)	1.02 (0.87–1.20)	1.16 (0.97–1.38)
No condom use during first sexual intercourse^c^	1.15 (0.78–1.68)	0.77 (0.67–0.87)***	0.72 (0.60–0.85)***	0.89 (0.74–1.06)
No condom use during most sexual intercourse^c^	1.13 (0.76–1.70)	0.76 (0.67–0.87)***	0.69 (0.57–0.84)***	0.87 (0.74–1.04)
Have had multiple sexual partners^c^	1.13 (0.82–1.56)	0.96 (0.88–1.06)	0.90 (0.79–1.03)	1.05 (0.93–1.18)
**Adverse sexual and reproductive health outcomes**		
Ever had unintended pregnancy^c^	0.70 (0.33–1.49)	0.76 (0.59–0.98)*	0.86 (0.59–1.25)	0.77 (0.54–1.10)
Ever had induced abortion^c^	0.62 (0.27–1.45)	0.83 (0.64–1.08)	0.77 (0.53–1.10)	0.74 (0.53–1.04)
Ever been diagnosed sexual transmitted infection	0.81 (0.44–1.46)	1.17 (0.93–1.48)	0.82 (0.59–1.14)	1.34 (1.01–1.76)*

† *Reference: students with siblings*.

a *Adjusted for age, sex, ethnicity, school type, average monthly expenditure, self-rated parent-child relationship, parents' highest educational attainments, tobacco consumption, alcohol consumption*.

b *Adjusted for age, hometown region, ethnicity, school type, average monthly expenditure, self-rated parent-child relationship, ever received sexuality education at school, parents' highest educational attainments, tobacco consumption, alcohol consumption*.

c *Excludes students who have never had sexual intercourse (included = 10,606). The details are showed in Supplementary Table S1*.

d *Use linear regression reporting coefficient*.

^*^p < 0.05, ^**^p < 0.01, ^***^p < 0.001.

Only-child students were more likely to agree with premarital sexual intercourse, if their hometown region was in an urban area (OR 1.34, 95% 1.27–1.41), and regardless of sex (males: OR 1.14, 95% CI 1.06–1.22; females: OR 1.47, 95% CI 1.38–1.57); sexual intercourse during university, if their hometown region was in an urban area (OR 1.39, 95% CI 1.32–1.46), and regardless of sex (males: OR 1.17, 95% CI 1.09–1.26; females: OR 1.50, 95% CI 1.40–1.58); one-night stand, if their hometown region was in an urban area (OR 1.17, 95% CI 1.08–1.27) and if they were female (OR 1.35, 95% CI 1.22–1.50); and multiple sexual partners, if their hometown region was in an urban area (OR 1.51, 95% CI 1.40 to 1.64), and regardless of sex (males: OR 1.24, 95% CI 1.11–1.38; females: OR 1.69, 95% CI 1.53–1.86).

However, only-child students were less likely to feel guilty for being sexually active, if their hometown region was in an urban area (OR 0.85, 95% 0.80–0.91), and regardless of sex (males: OR 0.90, 95% CI 0.82–0.99; females: OR 0.81, 95% CI 0.74–0.88).

Only-child students who were females and whose hometown region was in an urban area were more likely to have ever read erotic magazines or books (female: OR 1.27, 95% CI 1.20–1.35; urban: OR 1.16, 95% CI 1.10–1.21), and have ever search for pornographic information (female: OR 1.70, 95% CI 1.61–1.81; urban: OR 1.55, 95% CI 1.47–1.64) and sexual knowledge online (female: OR 1.63, 95% CI 1.53–1.73; urban: OR 1.47, 95% CI 1.40–1.55). Only-child students whose hometown region was in a rural area were more likely to have ever had casual sexual intercourse (OR 1.69, 95% CI 1.12–2.56), and those in urban area were less likely to have no condom use during first sexual intercourse (OR 0.77, 95% CI 0.67–0.87) and no condom use during most sexual intercourse (OR 0.76, 95% CI 0.67–0.87) compared to students with siblings, especially if they were males. Only-child students whose hometown region is in an urban area were at lower odds of having ever had an unintended pregnancy (OR 0.76, 95% CI 0.59–0.98), while female only-child students were more likely to ever have been diagnosed with STI (OR 1.34, 95% CI 1.01–1.76).

## Discussion

In this cohort of students, we found several main findings. Compared to students with siblings, only-child students have higher sexual knowledge, more liberal sexual attitudes, were more likely to seek sexual information, and have higher sexual behaviors but fewer adverse SRH outcomes. The results were influenced by sex and hometown region.

Our study found that only-child students have higher sexual-related knowledge scores compared to students with siblings. A possible reason is that because cultural norms in China inhibits discussion of sexual matters, especially for adolescents and unmarried youth, numerous Chinese students receive limited information on sex (25). Given that parents of only-child students are more likely to be more financially well-off, only-child students may have greater access to technology and the Internet (26), which provides them with additional sources of access to sexual knowledge (27). A study suggested that higher sexual knowledge is also associated with more liberal attitudes toward one-night stands and premarital sex (28). This is a possible explanation as to why only-child students tend to have more liberal sexual attitudes (29), be more sexually active, and have greater number of sexual partners (30).

We also found that only-child students were more likely to use condoms during sexual intercourse compared to students with siblings. Given that in this cohort, only-child students were more likely to have had parent-child discussion related to contraceptive use compared to those with siblings, they may be more aware of the need for contraceptive use during intercourse. Furthermore, only-child students were found to be more likely to actively search for sex-related information online (20). A survey conducted in Malaysia reported that respondents who searched for sex-related information online had greater intention to use condoms during intercourse compared to those who did not (31). A study conducted in Chinese college students also reported a positive relationship between exposure to porn and condom use (32). Given that a higher proportion of only-child students in this cohort of students, reported having had searched for sexual information and pornographic materials online, this may explain the greater use of condoms during sexual intercourse.

Compared to males, the differences between only-child students and students with siblings in sexual knowledge and attitudes were more significant in females. Females tend to have better communication skills and may therefore obtain greater sexual knowledge from friends and family (20). Previous research has indicated that the sexual attitudes and behaviors of children are associated with their mothers, as mothers were more likely to discuss sex-related topics with daughters (12). Besides, as a predominantly patriarchal society, and despite social revolutions over the recent decades, son preference still exists in China today. Therefore, male-biased parents with multiple children may allocate more resources to sons than daughters (33). However, in one-child families, females may be more similarly treated as to males because they are an only-child (26, 34). Several studies have supported this notion; only-child females reported better educational opportunities compared to females with siblings (35, 36).

Compared to females, the difference between only-child status with condom use during sexual intercourse were more significant in males. This is because in China, due to the concept of male superiority, females hold less power when negotiating safe sex (37). Studies have suggests that lack of control over sexual behaviors is associated with a lower likelihood of condom use among women aged 15–24 (38, 39). Furthermore, our findings indicate that only-child students were more likely to have parent-child discussion relevant to sexual behaviors and parent-child discussion relevant to contraception than students with siblings. This suggests that only-child students are more likely to be aware of the importance of condom use during sexual intercourse. Therefore, the difference in condom use during sexual intercourse is more significant between male only-child students and students with siblings.

Compared to rural areas, the difference between only-child students and students with siblings in sexual knowledge, attitudes, behaviors and SRH outcomes were more significant in urban areas. Only-child students living in urban areas may have better access to sexuality education as urban regions may have better educational opportunities and facilities. Furthermore, because the phenomenon of being left-behind is prevalent in rural regions, students from rural regions may lack social and economic support from parents (40). Studies have also consistently reported rural-urban inequalities in access to education due to the household registration (hukou) system in China (41, 42), which excludes rural residents from access to state-allocated resources (43). Therefore, students from rural areas tend to have fewer education opportunities, regardless of their only-child status. Given the low quality of sexuality education in rural regions, only-child may not be able to obtain good sexuality education, much less those with siblings. Urban regions were also found to have better sexuality education programs compared to sexuality education programs in rural areas (20). Therefore, the difference in sexual knowledge, attitudes, behaviors and SRH outcomes is smaller between only-child students and students with siblings in rural areas.

Overall, our study has several strengths. First, our study has a large sample size and given that our data was collected across various areas in China, it is nationally representative and allows for better generalizability of results. Second, our study reports on the difference in sex-related knowledge, attitudes, behaviors and SRH outcomes between only-child students and students with siblings stratified for sex and region in China, with the control of important covariates such as sexuality education from both school and family. Lastly, compared to existing literature, measures on only child status with seeking behaviors for sex-related information were also collected and analyzed, providing greater comprehensiveness.

Our study also has several limitations. First, causal inference cannot be drawn as this was a cross-sectional study. Second, there might be response bias as sex-related topics remains a taboo topic in China. However, we utilized an internet-based approach to minimize response bias. Third, as the study utilizes self-reporting, there may be recall bias although logic correction was employed to reduce the number of incorrect answers. Fourth, we were unable to control for covariates such as sibling sex and age composition, which were not collected in this survey.

Subsequent studies should investigate the relationship between sibling sex, number of siblings, and age gaps between siblings with sexual and reproductive health in students. It would be beneficial also, to study the effects of only-child status and SRH in later years, such as during adulthood given that the prevalence of sexual behaviors and therefore, adverse reproductive health outcomes, are relatively low in college students.

## Conclusion

Only-child students demonstrated higher sexual knowledge, more liberal sexual attitudes, and fewer risky sexual behaviors. Female students and students who resided in rural areas were more likely to demonstrate seeking behaviors for sex-related information both offline and online. Comprehensive sexual education for students should aim to better include females and students from rural areas by ensuring that sexuality education is accessible and available both offline and online. Public healthcare may also provide sexual health clinics offering subsidized consultations and contraceptives.

## Data Availability Statement

The raw data supporting the conclusions of this article will be made available by the authors, without undue reservation.

## Author Contributions

YL, XQ, SZ, and KT conceived the presented idea. SZ planned and carried out the simulations. SZ and YL took the lead in writing the manuscript with the support from JH and XQ. All authors discussed the results and contributed to the final manuscript.

## Conflict of Interest

The authors declare that the research was conducted in the absence of any commercial or financial relationships that could be construed as a potential conflict of interest.

## Publisher's Note

All claims expressed in this article are solely those of the authors and do not necessarily represent those of their affiliated organizations, or those of the publisher, the editors and the reviewers. Any product that may be evaluated in this article, or claim that may be made by its manufacturer, is not guaranteed or endorsed by the publisher.

## References

[1] Lu Y Zhang Y Cao X Wang C Wang Y Zhang M. Forty years of reform and opening up: China's progress toward a sustainable path. Sci Adv. (2019) 5:eaau9413. 10.1126/sciadv.aau9413 31457075 PMC6685713

[2] Chen L. Problematic pornography use in China. Curr Addict Reports. (2022) 9:80–5. 10.1007/s40429-022-00408-9 35433194 PMC8990487

[3] Cui Z Mo M Chen Q Wang X Yang H Zhou N. Pornography use could lead to addiction and was associated with reproductive hormone levels and semen quality: a report from the MARHCS Study in China. Front Endocrinol (Lausanne). (2021) 1:736384. 10.3389/fendo.2021.736384 34566897 PMC8461095

[4] Bearinger LH Sieving RE Ferguson J Sharma V. Global perspectives on the sexual and reproductive health of adolescents: patterns, prevention, and potential. Lancet. (2007) 369:1220–31. 10.1016/S0140-6736(07)60367-5 17416266

[5] Zhang K Beck EJ. Changing sexual attitudes and behaviour in China: implications for the spread of HIV and other sexually transmitted diseases. AIDS Care. (1999) 11:581–9. 10.1080/09540129947730 10755033

[6] Vuong QH Napier NK. Acculturation and global mindsponge: an emerging market perspective. Int J Intercult Relations. (2015) 49:354–67. 10.1016/j.ijintrel.2015.06.003

[7] Xie Y Wang X Mu Y Liu Z Wang Y Li X. Characteristics and adverse outcomes of Chinese adolescent pregnancies between 2012 and 2019. Sci Rep. (2021) 11:12508. 10.1038/s41598-021-92037-x 34131205 PMC8206124

[8] UNFPA, China Policy Brief Series. Ending Unintended Pregnancies among Chinese Youth by 2030. 2018. United Nations Population Fund (UNFPA) China. Available online at: https://www.semanticscholar.org/paper/Ending-Unintended-Pregnancies-among-Chinese-Youth/2a0bf69fe6f7bf332966b200228c7551a0fb64d1#:~:text=Ending%20Unintended%20Pregnancies%20among%20Chinese%20Youth%20by%202030,and%20greatly%20reduce%20the%20number%20of%20adolescent%20pregnancies%E2%80%9D

[9] Wang C. History of the Chinese family planning program: 1970–2010. Contraception. (2012) 85:563–9. 10.1016/j.contraception.2011.10.013 22176797

[10] Wen X. Effect of son preference and population policy on sex ratios at birth in two provinces of China. J Biosoc Sci. (1993) 25:509–21. 10.1017/S002193200002188X 8227099

[11] Bai FDr. Gu Baochang speaks out on son preference in China. China Popul Today. (1995) 12:27–8. 12290273

[12] Li HD Wang RRY. Analysis of the number and family structure of the only children since the family planning policy. Stat Decis. (2018) 34:99–104. 10.13546/j.cnki.tjyjc.2018.13.022 (in Chinese).

[13] Liu Y Jiang Q. Who benefits from being an only child? A study of parent–child relationship among Chinese Junior High School Students. Front Psychol. (2021) 11:608995. 10.3389/fpsyg.2020.608995 33488473 PMC7820425

[14] Li B Zhang H. Does population control lead to better child quality? Evidence from China's one-child policy enforcement. J Comp Econ. (2017) 45:246–60. 10.1016/j.jce.2016.09.004

[15] Chen C LeGates R Zhao M Fang C. The changing rural-urban divide in China's megacities. Cities. (2018) 81:81–90. 10.1016/j.cities.2018.03.017

[16] Lu Y Yeung WJJ Liu J Treiman DJ. Health of left-behind children in China: Evidence from mediation analysis. Chin J Sociol. (2019) 5:431–52. 10.1177/2057150X1987268534770230

[17] Zheng W Zhou X Zhou C Liu W Li L Hesketh T. Detraditionalisation and attitudes to sex outside marriage in China. Cult Health Sex. (2011) 13:497–511. 10.1080/13691058.2011.563866 21452090

[18] Shen YL Li SY. Occurrence of sexual behavior among college students in mainland China: a meta-analysis. Chin J Public Health. (2018) 34:142–7. 10.11847/zgggws1113830 (in Chinese).

[19] Yan X. A comparative study on the marriage outlook of only-child students and non - only -child college students. Heath Psychol J. (2003) 11:81–3. 10.13342/j.cnki.cjhp (in Chinese).19319804

[20] Li S Chen R Cao Y Li J Zuo D Yan H. Sexual knowledge, attitudes and practices of female undergraduate students in Wuhan, China: the only-child versus students with siblings. PLoS ONE. (2013) 8:e73797. 10.1371/journal.pone.0073797 24023905 PMC3762716

[21] Zou S Cao W Jia Y Wang Z Qi X Shen J. Sexual and reproductive health and attitudes towards sex of young adults in China. BMJ Sex Reprod Health. (2022) 48:e13–21. 10.1136/bmjsrh-2020-200766 33504512

[22] Falbo T Polit DF. Quantitative review of the only child literature: research evidence and theory development. Psychol Bull. (1986) 100:176. 10.1037/0033-2909.100.2.176

[23] Peate I. The effects of smoking on the reproductive health of men. Br J Nurs. (2005) 14:362–6. 10.12968/bjon.2005.14.7.17939 15924009

[24] Nespor K Scheansová A. Alcohol, tobacco and other addictive substances and reproductive health. Cas Lek Cesk. (2011) 15:339–43. 21751508

[25] Zhang L Li X Shah IH. Where do Chinese adolescents obtain knowledge of sex? Implications for sex education in China. Health Educ. (2007) 2:422–428+433. 10.3969/j.issn.1674-1889.2010.06.014 (in Chinese).

[26] Tsui M Rich L. The only child and educational opportunity for girls in urban China. Gend Soc. (2002) 16:74–92. 10.1177/089124320201600100527277601

[27] Hao Y Feng X. The influence of parent-child relation on the growth of onIy chiId. J Huazhong Univ Sci Technol (The Humanit Soc Sci Ed. (2002) 16:109–12. 10.19648/j.cnki.jhustss1980.2002.06.027 (in Chinese).

[28] Jin CC Zou H He S. Situation and relationship of college students' sexual knowledge and sexual attitude. Chin J Hum Sex. (2008) 17:10–4. 10.3877/j.issn.1672-1993.2008.02.003 (in Chinese).

[29] Dutt S Manjula M. Sexual knowledge, attitude, behaviors and sources of influences in Urban college youth: a study from India. Indian J Soc Psychiatry. (2017) 33:319. 10.4103/0971-9962.218602

[30] Jemmott LS Jemmott III JB. Sexual knowledge, attitudes, and risky sexual behavior among inner-city Black male adolescents. J Adolesc Res. (1990) 5:346–69. 10.1177/074355489053006

[31] Mohamad Shakir SM Wong LP Lim Abdullah K Adam P. Online STI information seeking behaviour and condom use intentions among young Facebook users in Malaysia. Health Promot Int. (2020) 35:1116–24. 10.1093/heapro/daz108 31665378

[32] Sun X Liu X Shi Y Wang Y Wang P Chang C. Determinants of risky sexual behavior and condom use among college students in China. AIDS Care. (2013) 25:775–83. 10.1080/09540121.2012.748875 23252705

[33] Echávarri R Husillos J. The missing link between parents' preferences and daughters' survival: the moderator effect of societal discrimination. World Dev. (2016) 78:372–85. 10.1016/j.worlddev.2015.10.037

[34] Hannum E Kong P Zhang Y. Family sources of educational gender inequality in rural China: a critical assessment. Int J Educ Dev. (2009) 29:474–86. 10.1016/j.ijedudev.2009.04.007 20161037 PMC2753976

[35] Lee MH. The one-child policy and gender equality in education in China: evidence from household data. J Fam Econ Issues. (2012) 33:41–52. 10.1007/s10834-011-9277-912290503

[36] Chen Y Ebenstein A Edlund L Li H. Girl adoption in China—a less-known side of son preference. Popul Stud (NY). (2015) 69:161–78. 10.1080/00324728.2015.1009253 25789605

[37] Yang Y Ye Y Yang Y Lin W Liang H CQ. Study of relationship between condom use consistently and at sexual debut among college students. Reprod Contracept. (2014) 34:955–60.24971287

[38] Varga CA. How gender roles influence sexual and reproductive health among South African adolescents. Stud Fam Plann. (2003) 34:160–72. 10.1111/j.1728-4465.2003.00160.x 14558319

[39] Pettifor AE Measham DM Rees H V Padian NS. Sexual power and HIV risk, South Africa. Emerg Infect Dis. (2004) 10:1996. 10.3201/eid1011.040252 15550214 PMC3328992

[40] Chen R Zhou L. Parental Migration and Psychological Well-Being of Children in Rural China. Int J Environ Res Public Health. (2021) 18:8085. 10.3390/ijerph18158085 34360378 PMC8345461

[41] Li L Li S Chen Y. Better city, better life, but for whom? : The hukou and resident card system and the consequential citizenship stratification in Shanghai City. Cult Soc. (2010) 1:145–54. 10.1016/j.ccs.2010.09.003

[42] Frazier MW. One Country, Two Societies: Rural-Urban Inequality in Contemporary China. Cambridge, MA: Harvard University Press (2010). p. 1219–21. 10.1017/S0021911810002378

[43] Chan KW. The Chinese household registration system and recent developments. Popul Dev Rev. (2010) 36:357–64. 10.1111/j.1728-4457.2010.00333.x 20734556

