# Supplementary material for: Difference in the Sexual and Reproductive Health of Only-Child Students and Students With Siblings, According to Sex and Region: Findings From the National College Student Survey
Source: Front Public Health. 2022 Jul 8;10:925626. doi: 10.3389/fpubh.2022.925626 (PMC9309253; doi:10.3389/fpubh.2022.925626)
Supplement: Supplementary file 1 [file Table_1.pdf]

Supplementary Material

TABLE S1— Socio-demographic characteristics of only-child students and students with siblings who ever had sexual intercourse.

	Total (N = 10,606)	Students with siblings (n = 6,075)	Only-child students (n = 4,531)	*p*-value
	n (%)	n (%)	n (%)	
**Age (years), Mean ± SD**	20.53± 1.49	20.41 ± 1.48	20.68 ± 1.49	<0.0001
**Sex**				0.002
Male	4,458(42.03)	2,474 (40.72)	1,984(43.79)	
Female	6,148 (57.97)	3,601 (59.28)	2,547 (56.21)	
**Ethnicity**				<0.0001
Han	9,528 (89.84)	5,353 (88.12)	4,175 (92.14)	
Others	1,078 (10.16)	722 (11.88)	356 (7.86)	
**Hometown region**				<0.0001
Rural	1,400 (13.20)	1,209 (19.90)	191 (4.22)	
Urban/suburban	9,206 (86.80)	4,866 (80.10)	14,340 (95.78)	
**School type**				<0.0001
College	2,286 (21.55)	1,666 (27.42)	620 (13.68)	
University	8,320 (78.45)	4,409 (72.58)	3,911 (86.32)	
**Average monthly expenditure (RMB)**				<0.0001
0-999	581 (5.48)	451 (7.42)	130 (2.87)	
1000-1999	4,676 (44.09)	3,158 (51.98)	1,518 (33.50)	
⩾2000	5,349 (50.43)	2,466 (40.59)	2,883 (63.63)	
**Ever received sexuality education at school**	5,559 (52.41)	3,287 (54.11)	2,272 (50.14)	<0.0001
**Self-rated parent-child relationship (0-10), Mean ± SD**				
Fathers	6.72 ± 2.46	6.73 ± 2.44	6.70 ± 2.49	0.426
Mothers	7.54 ± 2.02	7.67 ± 2.02	7.45 ± 2.14	<0.0001
**Parental highest educational attainments**				<0.0001
Primary school and below	936 (8.83)	817 (13.45)	119 (2.63)	
Middle school	2,928 (27.61)	2,299 (37.84)	629 (13.88)	
High school	3,018 (28.46)	1,712 (28.18)	1,306 (28.82)	
College and above	3,724 (35.11)	1,247 (20.53)	2,477 (54.67)	
**Parent-child discussion relevant to sexual behaviors**				<0.0001
Never	6,860 (64.68)	4,275 (70.37)	2,585 (57.05)	
Ever	3,746 (35.32)	1,800 (29.63)	1,946 (42.95)	
**Parent-child discussion relevant to contraception**				<0.0001
Never	7,199 (67.88)	4,513 (74.29)	2,686 (59.28)	
Ever	3,407 (32.12)	1,562 (25.71)	1,845 (40.72)	
**Tobacco consumption**				0.029
Ever	4.381 (69.21)	4,153 (68.36)	3,187 (70.34)	
Never	3,266 (30.79)	1,922 (31.64)	1,344 (29.66)	
**Alcohol consumption**				<0.0001
Ever	4,381 (41.31)	2,688 (44.25)	1,693 (37.36)	
Never	6,225 (58.69)	3,387 (55.75)	2,838 (62.64)	
